# Identification and application of an exocarp-preferential promoter for genetic engineering of tomato fruit

**DOI:** 10.1093/hr/uhae035

**Published:** 2024-01-31

**Authors:** Xue-Ming Ruan, Xiangyu Xiong, Jian-Feng Li

**Affiliations:** State Key Laboratory of Biocontrol, Guangdong Provincial Key Laboratory of Plant Resources, School of Life Sciences, Sun Yat-sen University, Guangzhou 510275, China; State Key Laboratory of Biocontrol, Guangdong Provincial Key Laboratory of Plant Resources, School of Life Sciences, Sun Yat-sen University, Guangzhou 510275, China; State Key Laboratory of Biocontrol, Guangdong Provincial Key Laboratory of Plant Resources, School of Life Sciences, Sun Yat-sen University, Guangzhou 510275, China

## Abstract

Tomato (*Solanum lycopersicum*) is a globally cultivated crop with great economic value. The exocarp determines the appearance of tomato fruit and protects it from various biotic and abiotic challenges at both pre-harvest and post-harvest stages. However, no tomato exocarp-specific promoter is currently available, which hinders exocarp-based genetic engineering. Here, we identified by RNA sequencing and reverse transcription-quantitative PCR analyses that the tomato gene *SlPR10* (*PATHOGENESIS RELATED 10*) was abundantly and predominantly expressed in the exocarp. A fluorescent reporter expressed by a 2087-bp *SlPR10* promoter (*pSlPR10*) was mainly detected in the exocarp of transgenic tomato plants of both Ailsa Craig and Micro-Tom cultivars. This promoter was further utilized for transgenic expression of *SlANT1* and *SlMYB31* in tomato, which are master regulators of anthocyanin and cuticular wax biosynthesis, respectively. *pSlPR10*-driven *SlANT1* expression resulted in anthocyanin accumulation in the exocarp, conferring gray mold resistance and extended shelf life to the fruit, while *SlMYB31* expression led to waxy thickening in the fruit skin, delaying water loss and also extending fruit shelf life. Intriguingly, *pSlPR10* and two other weaker tomato exocarp-preferential promoters exhibited coincided expression specificities in the gynophore of transgenic *Arabidopsis* (*Arabidopsis thaliana*) plants, providing not only an inkling of evolutionary homology between tomato exocarp and *Arabidopsis* gynophore but also useful promoters for studying gynophore biology in *Arabidopsis*. Collectively, this work reports a desirable promoter enabling targeted gene expression in tomato exocarp and *Arabidopsis* gynophore and demonstrates its usefulness in genetic improvement of tomato fruit quality.

## Introduction

Tomato (*Solanum lycopersicum*) is one of the most important crops in the world owing to its broad planting areas, fast growth, and relatively high yield. With rich nutritional value, tomato fruit not only can be consumed fresh but also can be processed into various commercial products, such as tomato juice and ketchup, after peel and seed removal [1]. In fundamental research, as tomato is readily amenable to genetic transformation and gene editing [2] and possesses diversified germplasm collections [3], it represents a commonly used model species [4], particularly for studying fruit development and ripening. In addition, tomato fruit contains large amounts of metabolites and many basic biosynthetic pathways that can be enhanced by modulating the activity of endogenous transcription factors or be rewired by introducing exogenous metabolic enzymes, making it an excellent chassis for synthetic biology [5, 6].

Promoter plays a central role in determining the strength and spatiotemporal specificity of gene expression, which in turn poses impact on organismal trait formation [7]. Both transgenic and synthetic biology endeavors in tomato require efficient and well-tuned promoters for tight regulation of gene expression. Two major types of promoters, namely constitutive promoters and tissue-specific promoters, have been utilized for controlling the expression of a gene of interest (GOI) in tomato. Constitutive promoters exhibit robust activity in expressing the GOI across nearly all tissue types and developmental stages [8]. The most frequently used constitutive promoter is the cauliflower mosaic virus *35S* promoter, which has been widely employed to drive strong expression of GOIs in tomato for trait improvement. A tomato ubiquitin (*Ubi1–1*) promoter has also been explored for constitutive expression of a transgene in tomato [9]. However, the *35S* promoter-driven transgene expression is prone to transcriptional silencing in tomato [10]. Moreover, constitutive promoter-driven high expression of GOIs in the whole plant can result in unnecessary resource and energy consumption in untargeted tissues and may induce pleiotropic genetic perturbation or even deleterious effects on plant fitness [8]. For instance, the *35S* promoter-driven transgenic expression of *SlANT1*, which encodes a MYB transcription factor regulating anthocyanin biosynthesis [11], led to anthocyanin overaccumulation throughout the entire tomato plant and inhibition of plant growth [12].

In contrast to constitutive promoters, tissue-specific promoters only drive the expression of GOIs in intended plant tissues at defined developmental stages, thereby reducing physiological costs associated with GOI expression and minimizing potential adverse effects on plant growth. In tomato, fruit-specific promoters have attracted most attention due to their paramount importance in fundamental and biotechnological research. To date, many promoters have been isolated and characterized to show activities in multiple tissues of tomato fruit. Among them, the promoters of *2A11*, *LYCes;Ppc2*, *LA22CD07*, and *SlHDC-A* are effective during several fruit developmental stages [13–16], whereas those of *PG*, *E8*, *α-Man*, and *RIP1* are specific to the ripening process [15–20] and the *Tfm5* promoter seems to be active exclusively at immature stages [21]. In addition, two groups have developed versatile vector systems harboring a new collection of fruit-specific promoters, including *PPC2*, *TPRP*, *PNH*, *PLI*, *PFF*, *PHD*, and *PSN*, to facilitate targeted gene expression or silencing in tomato fruit with different spatiotemporal characteristics [22, 23]. The *PPC2* promoter is mainly active in the pericarp, placenta, and gel of young growing fruits. The *TPRP* promoter is highly expressed in all tissues of young fruits but exhibits dramatically reduced activity in mature green and ripe fruits. The *PNH* promoter drives high expression throughout all tissues in the developing fruits. The expression of *PLI* promoter is predominantly restricted to the outer pericarp at early developmental phases but spreads to the whole fruit and reaches a maximum at the ripe stage. The *PFF* promoter-driven expression displays a biphasic pattern, one localized to the central collumela at 12–18 days post-anthesis and the other covering all fruit tissues at the ripe stage. The *PHD* promoter enables transgene expression at moderate levels throughout the entire fruit during all developmental stages. The *PSN* promoter is only active in the central collumela and placenta at ripening stages. In addition to endogenous promoters, heterologous promoters provide an alternative source of fruit-specific promoters in tomato. For example, the potato (*Solanum tuberosum*) *agpB1* promoter can be expressed in the placenta and pericarp of tomato fruit [24], while the apple (*Malus pumila* Mill.) *ACO* or *PG* promoter can confer ripening-specific expression in tomato fruit [25].

Of note, the majority of characterized fruit-specific promoters are active across multiple cell types of tomato fruit. It is highly desirable to identify novel promoters with restricted cell type specificity to fulfill more precise genetic engineering in the fruit. One of such cell types with a specific promoter in demand is the exocarp, which consists of the cuticle and epidermal cells forming the skin of tomato fruit. The exocarp not only serves as a vital physical barrier protecting fruit against diverse biotic and abiotic stresses at both pre-harvest and post-harvest stages, but also influences the appearance, mechanical integrity, and shelf life of ripe fruit [26, 27]. Meanwhile, as a non-edible portion of ripe fruit, the exocarp is estimated to account for 56% of dried tomato pomace produced in the food industry [28], making it a potentially useful synthetic biology chassis that can be recycled from tomato peel wastes for extracting synthesized high-value compounds. However, there is currently no tomato exocarp-specific promoter available, which limits exocarp-based genetic engineering and synthetic biology.

Pathogenesis related (PR) proteins are a collection of unrelated low-molecular-weight proteins that perform diversified protective roles in plants under various biotic and abiotic stresses. Based on the sequence, structural, or biochemical similarity, PR proteins have been categorized into 17 families [29]. The *PR10* genes form a multigene superfamily encoding PR proteins of the class 10. Although more than a hundred of *PR10* members have been identified from over 70 plant species, their physiological functions remain poorly understood [30]. Evidence for a possible role of PR10 proteins in plant defense comes from the observations that some representatives have antifungal activities as a ribonuclease [31, 32]. Interestingly, in this study attempting to identify exocarp preferentially expressed genes in tomato, we came across *SlPR10*, which encodes a putative PR10 protein with unknown function. *SlPR10* was found to be abundantly and predominantly expressed in the exocarp of both mature green and red ripe fruits. By using the *SlPR10* promoter, we successfully engineered anthocyanin and cuticular wax biosynthesis in the exocarp of transgenic tomato plants, which helped to improve multiple traits of tomato fruit.

## Results

### Comparative transcriptomic analysis of tomato exocarp, mesocarp, and leaves uncovers exocarp preferentially expressed genes

**Figure 1:**
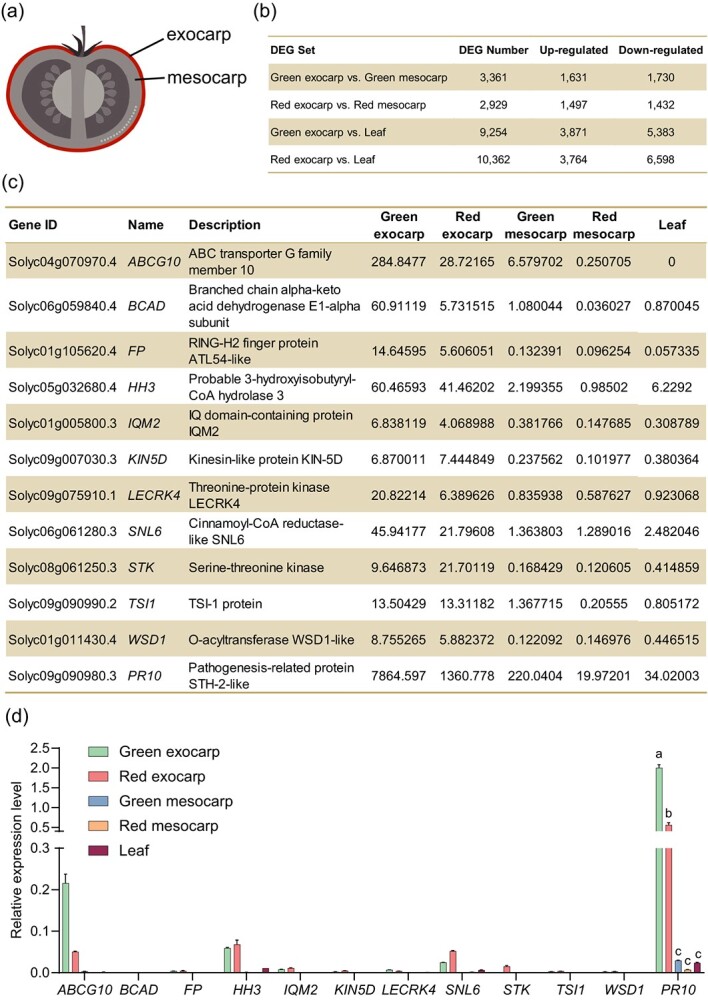
Comparative transcriptomic profiling and RT-qPCR validation reveal exocarp preferentially expressed genes in tomato. (**a**) Diagram of tomato fruit cross-section showing the exocarp and mesocarp. (**b**) Numbers of differentially expressed genes (DEGs) between the exocarp and mesocarp or pooled leaves in RNA-seq. Green, 30 days post-anthesis. Red, 7 days post-breaker. Indicated tissues were obtained from tomato cv. Ailsa Craig (AC) and RNA-seq analyses were conducted with two biological replicates. (**c**) Summary of 12 candidate genes selected as exocarp preferentially expressed genes for RT-qPCR validation. The numbers indicate the FPKM (fragments per kilobase of transcript per million fragments mapped). (**d**) RT-qPCR evaluation of expression levels of the 12 candidate genes in the exocarp, mesocarp, and pooled leaves. Data are shown as means ± SD of three biological replicates. For each gene, columns from left to right correspond to mature green exocarp, red ripe exocarp, mature green mesocarp, red ripe mesocarp, and pooled leaves from both developmental stages, respectively. *SlACTIN* was used as a reference gene to normalize the relative expression level. Different letters for *PR10* indicate significant differences with *P* < 0.05 (two-way ANOVA with Tukey’s multiple comparisons test).

The aim of this study was to identify an exocarp-specific promoter enabling targeted trait improvement in the exocarp of tomato fruit. For this purpose, we started with RNA-sequencing (RNA-seq) analysis for five different tissues of tomato cv. Ailsa Craig (AC), namely the exocarp or mesocarp (Fig. 1a) of mature green fruits at 30 days post-anthesis (dpa) or red ripe fruits at 7 days post-breaker (dpb) and pooled leaves. Comparison of transcriptomic profiles revealed 3,361 differentially expressed genes (DEGs) between the mesocarp and exocarp at the green mature stage and 2,929 DEGs at the red ripe stage (Fig. 1b). The Kyoto Encyclopedia of Genes and Genomes (KEGG) analysis highlighted that the DEGs between the exocarp and mesocarp at both stages were overrepresented by genes engaged in plant-pathogen interactions (Fig. S1a and b, see online supplementary material), which was in agreement with an expected role of the exocarp in shielding the internal tissues of fruit from pathogen infection. On the other hand, the exocarp of mature green or red ripe fruit exhibited 9,254 and 10,362 DEGs, respectively, in comparison with leaves (Fig. 1b). Of note, an overlapping subset of 755 genes were found to be differentially expressed in the exocarp when compared to the mesocarp and leaves (Fig. S2, see online supplementary material), suggesting that the transcriptional changes of these genes are likely associated with exocarp development or physiology.

**Figure 2:**
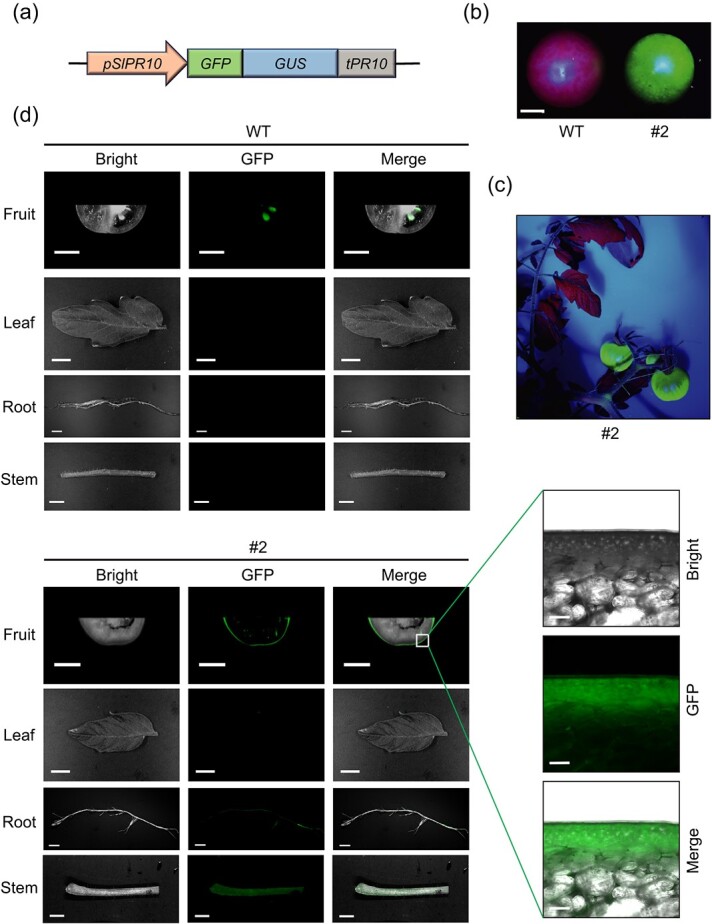
The *SlPR10* promoter enables exocarp-preferential expression of a reporter gene in transgenic AC tomato. (**a**) Diagram of the transgenic expression cassette containing the *SlPR10* promoter (*pSlRP10*), a *GFP-GUS* fusion reporter gene, and the *SlPR10* terminator (*tPR10*). (**b**) Transgenic fruit showing GFP fluorescence. A representative fruit from the transgenic line #2 was illuminated by a fluorescence flashlight. WT, wild type. Scale bar = 1 cm. (**c**) Transgenic plant illuminated by a fluorescence flashlight. (**d**) Comparison of GFP fluorescence in the mature green fruit (cross-section), leaf, root, and stem between transgenic and WT plants. Scale bar = 1 cm. The images on the right show a magnified view (scale bar = 80 μm) of the boxed region in the transgenic fruit.

On the basis of the RNA-seq data, we focused on 12 exocarp preferentially expressed candidate genes (Fig. 1c) according to two criteria: these genes showed undetectable or low expression in leaves, with the FPKM (fragments per kilobase of transcript per million fragments mapped) below 50; the transcript levels of these genes in the exocarp at both mature green and red ripe stages must be at least tenfold higher than those in the mesocarp. We further validated the relative expression levels of these genes in the exocarp, mesocarp, and leaves by reverse transcription-quantitative PCR (RT-qPCR) using the tomato housekeeping gene *SlACTIN* as a reference gene. The results indicated *SlPR10* (Solyc09g090980.3), *SlABCG10* (Solyc04g070970.4), and *SlHH3* (Solyc05g032680.4) as the top three exocarp preferentially expressed genes (Fig. 1d). Although the expression of *SlABCG10* appeared to be most restricted to the exocarp, we selected *SlPR10* to work with because this gene exhibited an extremely high expression level in the exocarp, reaching more than tenfold of that of *SlABCG10* and twofold of that of *SlACTIN* (Fig. 1d). To better understand the tissue specificity of *SlPR10* expression, we also investigated its expression in the root and stem of AC plants. The results demonstrated that *SlPR10* was barely expressed in the root, whereas a moderate level of expression (i.e. ~10% relative to that in the exocarp) was detected in the stem (Fig. S3a, see online supplementary material). These results confirmed that *SlPR10* is abundantly and predominantly expressed in the exocarp of tomato plants.

### The *SlPR10* promoter enables exocarp-preferential expression of a reporter gene in transgenic tomato

To test whether the *SlPR10* promoter is useful as an exocarp-preferential promoter for transgene expression, we generated transgenic tomato cv. AC plants, in which a fusion reporter gene encoding GFP and β-glucuronidase (GUS) was placed between a 2087-bp *SlPR10* promoter (*pSlPR10*) and the *SlPR10* terminator (*tPR10*) (Fig. 2a). The fusion of GUS to GFP could prevent the free GFP from moving to neighboring tissues through the plasmodesmata [33], which otherwise would confound the determination of tissue specificity of *GFP* expression. When illuminated by a fluorescence flashlight, the fruits of transgenic plants expressing *pSlPR10::GFP-GUS* were found to emit strong GFP fluorescence, whereas the rest parts of transgenic plants displayed undetectable or little GFP fluorescence (Fig. 2b and c). To verify that the GFP fluorescence indeed came from the exocarp, cross-sections of transgenic fruits at 30 dpa were examined using a fluorescence imaging system and a fluorescence microscope sequentially. Although the seeds of wild-type (WT) control fruit somehow showed autofluorescence, only the exocarp of transgenic fruit produced intense GFP fluorescence (Fig. 2d). GFP signal could also be weakly detected in the stem but hardly detectable in the mesocarp, leaf, and root (Fig. 2d). The observed reporter activity was in accordance with the transcript levels of *SlPR10* determined earlier by RT-qPCR (Fig. 1d; Fig. S3a, see online supplementary material), suggesting that the 2087-bp *pSlPR10* is sufficient for strong and predominant transgene expression in the exocarp of tomato plants.

We noted that the 2087-bp *pSlPR10* in the tomato cv. AC was identically present in the tomato cv. Heinz 1706, the genome of which has recently been sequenced [34]. Moreover, we cloned and confirmed by Sanger sequencing that the tomato cv. Micro-Tom, a miniature tomato variety highly useful in basic research, also possesses the same 2087-bp *pSlPR10*. In addition, according to a spatiotemporal transcriptome mapping study using tomato cv. M82 fruits [35], *SlPR10* was also found to be highly expressed in the exocarp but not in the mesocarp (Fig. S3b, see online supplementary material). These findings indicated potentially conserved transcriptional regulation of *SlPR10* in different tomato varieties. To pinpoint whether *pSlPR10* also functions as an exocarp-preferential promoter in other tomato cultivars, we constructed transgenic Micro-Tom plants expressing *pSlPR10::GFP-GUS*. Similar to what has been observed in transgenic AC plants, transgenic Micro-Tom plants also showed very strong GFP fluorescence in the exocarp (Fig. S4, see online supplementary material), whereas elevated GFP fluorescence was seen in the mesocarp and stem. These results implied that *pSlPR10* is potentially useful for exocarp-preferential transgene expression in multiple tomato varieties.

### *pSlPR10*-driven transgenic expression of *SlANT1* produces purple-skinned tomato fruit

Next, we sought to demonstrate the application of *pSlPR10* in genetic improvement of tomato fruit quality. Because the exocarp functions as the physical shield of tomato fruit and anthocyanins can help protect plant cells against various biotic and abiotic stresses [27, 36–38], we hypothesized that anthocyanin accumulation in the exocarp should be sufficiently effective for enhancing stress resistance of tomato fruit. Therefore, we employed *pSlPR10* for transgenic expression of *SlANT1* [11], which was anticipated to promote anthocyanin biosynthesis in the exocarp.

**Figure 3:**
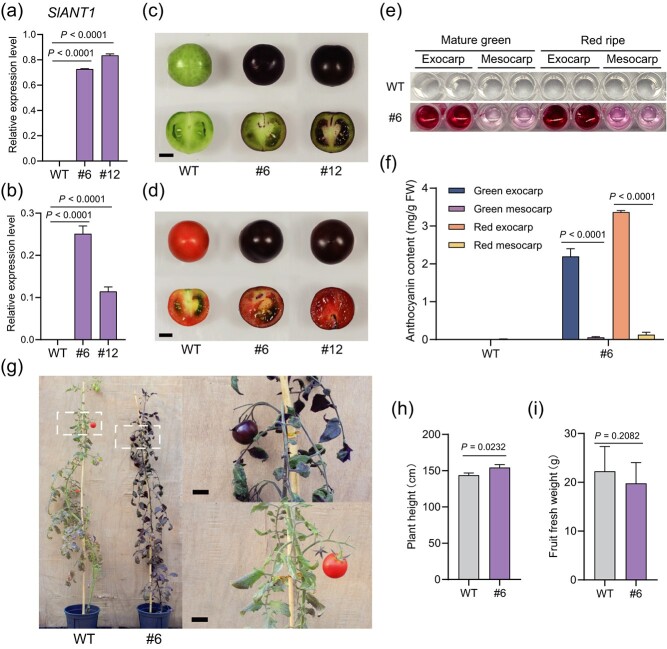
*pSlPR10*-driven transgenic expression of *SlANT1* produces tomato fruit with anthocyanin enrichment in the exocarp. (**a**, **b**) RT-qPCR analyses revealed high levels of *SlANT1* transcripts in the exocarp of transgenic AC fruits at the mature green (**a**) or red ripe (**b**) stage. Data are shown as means ± SD of three biological replicates. *SlACTIN* was used as a reference gene to normalize the relative expression level. (**c**, **d**) The skin of transgenic fruits at the mature green (**c**) or red ripe (**d**) stage exhibited a dark purple appearance. Scale bar = 1 cm. (**e**, **f**) Anthocyanin overaccumulation in the exocarp of transgenic fruits seen by naked eyes (**e**) or measured based on absorbance values (**f**). The anthocyanin content was measured as (A535-A650)/g fresh weight and further expressed as mg/g fresh weight according to the standard curve generated using petunidin-3-(p-coumaryl)-rutinoside-5-glucoside as a reference standard. Data are presented as means ± SD (*n* = 3). For indicated plants, columns from left to right correspond to mature green exocarp,mature green mesocarp, red ripe exocarp, and red ripe mesocarp, respectively. (**g**) Transgenic plant grown in a greenhouse under natural light conditions displayed normal growth. The images on the right show a magnified view of the boxed region in the transgenic or WT plant. Scale bar = 2 cm. (**h**) Quantification and comparison of heights between two-month-old transgenic and WT plants. Data are presented as means ± SD (*n* = 3). (**i**) Quantification and comparison of fruit fresh weights between transgenic and WT plants. Data are presented as means ± SD (*n* = 10). In (**a**), (**b**), (**f**), (**h**), and (**i**), statistical analyses were conducted using two-tailed Student’s *t*-test.

We noted that *SlANT1* expression was completely absent in the exocarp of tomato cv. AC fruit at the mature green (30 dpa) or red ripe (7 dpb) stage (Fig. 3a and b), which was in agreement with a recent study [39]. By contrast, in representative transgenic AC plants expressing *pSlPR10::SlANT1*, abundant transcripts of *SlANT1* could be detected by RT-qPCR in the exocarp, where its expression levels corresponded to ~80% of that of *SlACTIN* at the mature green stage (Fig. 3a) and then decreased a bit at the red ripe stage (Fig. 3b). Consistent with a positive regulatory role of *SlANT1* in anthocyanin biosynthesis, anthocyanin enrichment could be visualized in the exocarp at as early as 10 dpa and gradually reached a maximum at 30 dpa. Impressively, the skin of both mature green and red ripe fruits in transgenic lines with robust *pSlPR10::SlANT1* expression exhibited a dark purple appearance, while the flesh was only colored in light purple (Fig. 3c and d). Because anthocyanins are water-soluble, light purple coloration of the flesh in transgenic fruit might be an inevitable consequence of anthocyanin overflowing from the exocarp. We further quantified the anthocyanin content in the transgenic fruits using petunidin-3-(p-coumaryl)-rutinoside-5-glucoside, a predominant form of endogenous anthocyanins [40], as a reference standard. The highest anthocyanin concentration in the exocarp of transgenic fruits at the mature green or red ripe stage averaged around 2.2 and 3.5 mg per g fresh weight, respectively (Fig. 3e and f). By contrast, there were only modest levels of anthocyanins in the mesocarp of transgenic fruits at both stages, while anthocyanins were completely undetectable in the WT fruits (Fig. 3e and f).

In a previous study [12], the *35S* promoter-driven transgenic expression of *SlANT1* in tomato resulted in dwarf plants due to anthocyanin overaccumulation. Although the transgenic AC plants expressing *pSlPR10::SlANT1* in a greenhouse under natural light conditions somehow showed visible anthocyanin accumulation in vegetative tissues, these plants displayed comparable growth rates and heights as WT plants (Fig. 3g and h; Fig. S5, see online supplementary material). Also, there was no significant difference in the fruit sizes between transgenic and WT fruits (Fig. 3g and i). These results suggested that modest levels of anthocyanin accumulation in vegetative organs do not significantly inhibit plant growth.

To explore whether transgenic expression of *pSlPR10::SlANT1* could lead to purple-skinned fruit in other tomato varieties, we also generated transgenic tomato plants expressing *pSlPR10::SlANT1* in the Micro-Tom background. Transgenic Micro-Tom fruits also exhibited strong purple coloration of the skin at both mature green and red ripe stages (Fig. S6a and b, see online supplementary material). Whereas the anthocyanin content in the exocarp of WT (Micro-Tom) fruits at the mature green or red ripe stage was below the detection limit, transgenic fruits at these developmental stages contained anthocyanins at as high as 1.3 and 2.6 mg per g fresh weight, respectively (Fig. S6c and d, see online supplementary material). Anthocyanin accumulation to up to 0.3 mg per g fresh weight was also detected in the mesocarp (Fig. S6a–d, see online supplementary material), probably due to the increased activity of *pSlPR10* in the mesocarp of tomato cv. Micro-Tom (Fig. S4, see online supplementary material). Noteworthily, shading or complete darkness for 10 days exerted no obvious effect on the degree of fruit purple coloration (Fig. S7a and b, see online supplementary material). In consistent with these observations, when protoplasts isolated from the transgenic AC plants expressing *pSlPR10::GFP-GUS* were divided into two equal aliquots, one incubated under the light for 6 h and the other incubated in the dark, we failed to see a dramatic increase of GFP-GUS abundance upon light exposure relative to the group in the dark (Fig. S7c and d, see online supplementary material). These results together indicated that the *pSlPR10* activity is very likely light-independent.

Once more, although moderate levels of anthocyanin accumulation could be observed in leaves of transgenic Micro-Tom plants grown in a greenhouse, no growth inhibition or dramatic change of fruit sizes was noticed in transgenic plants when compared to WT plants (Fig. S8a and b, see online supplementary material). Taken together, these findings underscored the general usefulness of *pSlPR10* in exocarp-based genetic engineering of different tomato varieties.

### Purple-skinned tomato fruit exhibits gray mold resistance and improved shelf life

**Figure 4:**
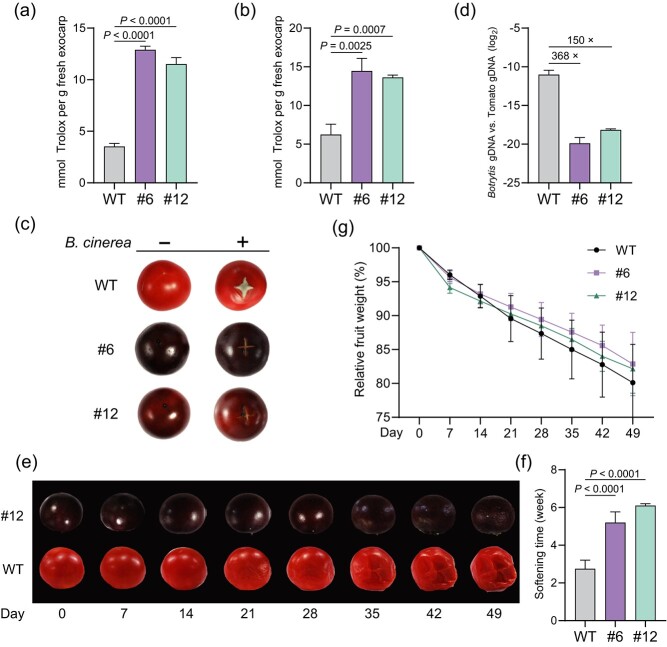
*pSlPR10*-driven transgenic expression of *SlANT1* confers gray mold resistance and extended shelf life to tomato fruit. (**a**, **b**) Transgenic AC fruits displayed increased antioxidant capacity relative to WT fruits at the mature green (**a**) or red ripe stage (**b**). Data are presented as means ± SD. (**c**) Transgenic fruits exhibited enhanced *Botrytis cinerea* resistance. Fruits harvested at 7 dpb were inoculated with 5,000 spores of *B. cinerea* at the center of a 1-cm long and 1-mm deep cross-shaped wound for 3 days. (**d**) Quantification of *B. cinerea* growth on the fruit surface at 3 days post inoculation. *B. cinerea* gDNA and tomato gDNA were quantified by qPCR based on the *Bc-Cutin* and *Sl-Actin* genes, respectively. Data are presented as means ± SD. (**e**) Transgenic fruits showed delayed softening and collapse compared to WT fruits. Fruits at the breaker stage were stored at room temperature for 7 weeks and were photographed every week. (**f**) Quantification of the fruit softening time. Fruit softening was visually assessed. Data are presented as means ± SD. (**g**) No significant difference in the water loss rate was detected between transgenic and WT fruits. Data are presented as means ± SD. In (**a**), (**b**), (**d**), (**f**), and (**g**), three randomly selected transgenic or WT fruits were assayed. All statistical analyses were conducted using two-tailed Student’s *t*-test.

Anthocyanins are known as antioxidants conferring enhanced disease resistance and extended shelf life to transgenic tomato fruit with fortified anthocyanin biosynthesis [27, 36, 37]. Consistently, as determined by the Trolox equivalent antioxidant capacity (TEAC) assay, the total antioxidant capacity of purple-skinned AC fruits at either the mature green or red ripe stage was substantially higher than that of WT fruits at the same developmental stage (Fig. 4a and b).

To assess whether anthocyanin accumulation by targeted *SlANT1* expression in the exocarp could boost disease resistance, purple-skinned ripe AC fruits were challenged with the gray mold pathogen *Botrytis cinerea*. Due to weak infection of WT control fruits by spray-inoculated *B. cinerea* spores in our preliminary experiments, we created a 1-cm long and 1-mm deep cross-shaped wound on the fruit and inoculated *B. cinerea* spores at the center of the wound. At 3 days post inoculation, the WT fruits supported massive fungal growth around the wound site, whereas the purple-skinned fruits showed no obvious disease symptom (Fig. 4c). The qPCR-based quantification of fungal biomass revealed 150- to 368-fold of fungal growth in WT fruits relative to purple-skinned fruits (Fig. 4d), which validated greatly potentiated *B. cinerea* resistance in purple-skinned fruits. Given that some PR10 proteins in other plant species have been shown as potential players in antifungal immunity [31, 32], we asked whether the *pSlPR10* itself might be induced upon *B. cinerea* infection. When *B. cinerea* spores were inoculated on the leaf surface of transgenic AC plants expressing *pSlPR10::SlANT1*, we did not notice a significant increase of SlANT1 abundance after 6 h treatment relative to the mock treatment (Fig. S9a and b, see online supplementary material).

**Figure 5:**
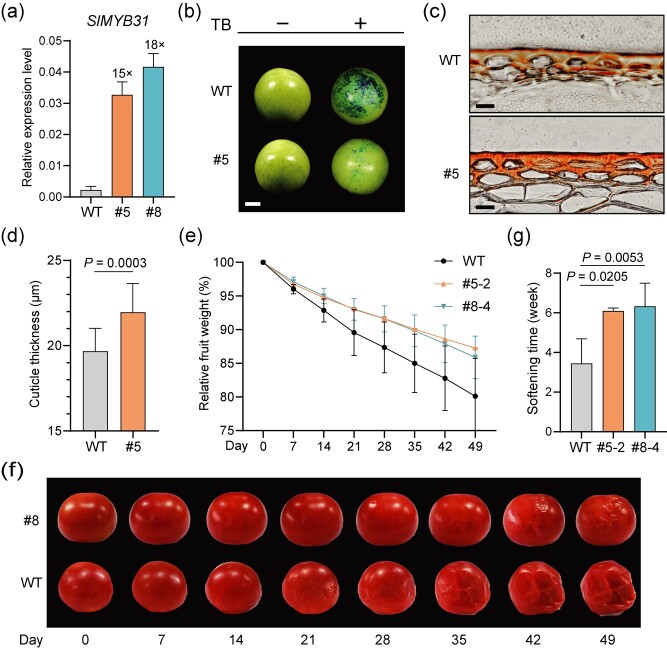
*pSlPR10*-driven transgenic expression of *SlMYB31* produces tomato fruits with delayed water loss and extended shelf life. (**a**) RT-qPCR analysis revealed increased *SlMYB31* transcript levels in the exocarp of transgenic mature green fruits (30 dpa) relative to WT fruits. Data are shown as means ± SD. *SlACTIN* was used as a reference gene to normalize the relative expression level. (**b**) Toluidine blue (TB) staining indicated reduced water permeability for transgenic mature green fruits. Scale bar = 1 cm. (**c**) Sudan III staining indicated increased cuticular wax thickness for transgenic mature green fruits. Scale bar = 20 μm. (**d**) Quantification of the cuticular wax thickness in transgenic and WT fruits. Data are shown as means ± SD. Five randomly selected fruits were assays and 15 measurements were performed for each fruit. (**e**) Transgenic fruits exhibited slower water loss relative to WT fruits during a 7-week storage period. Data are presented as means ± SD. (**f**) Transgenic fruits showed delayed softening and collapse compared to WT fruits. Fruits at the breaker stage were stored at room temperature for 7 weeks and were photographed every week. (**g**) Quantification of the fruit softening time. Fruit softening was visually assessed. Data are presented as means ± SD. In (**a**), (**b**), (**e**), and (**g**), three randomly selected transgenic or WT fruits were assayed. All statistical analyses were conducted using two-tailed Student’s *t*-test.

**Figure 6:**
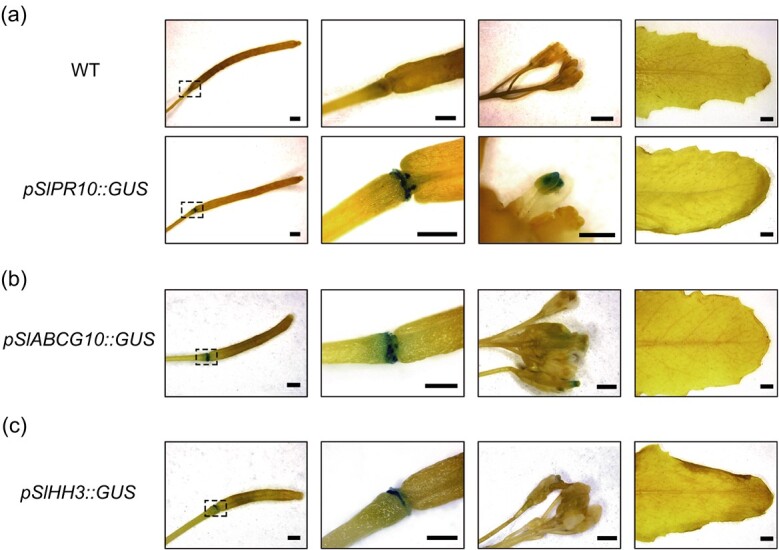
Three tomato exocarp-preferential promoters exhibit coincided activities in the gynophore of *Arabidopsis*. (**a**) Transgenic plants expressing *pSlPR10::GUS* showed strong GUS activity in the gynophore and weak activity in the anthers. (**b**) Transgenic plants expressing *pSlABCG10::GUS* showed strong GUS activity in the gynophore and weak activity in the stigma. (**c**) Transgenic plants expressing *pSlHH3::GUS* shows strong GUS activity in the gynophore. In (**a**)–(**c**), the second image shows a magnified view of the boxed region in the first image. Note that no GUS activity was detected in *Arabidopsis* leaves in all cases. Scale bar = 2 mm.

To evaluate the potential impact of anthocyanin accumulation in the exocarp on the shelf life of tomato fruits, storage tests using detached AC fruits at the breaker stage were conducted at room temperature over a period of 7 weeks. The purple-skinned fruits demonstrated delayed fruit softening and collapse when compared to WT fruits (Fig. 4e and f). By contrast, there was no significant difference in the water loss rates between purple-skinned and WT fruits (Fig. 4g). Collectively, these results demonstrated that *pSlPR10*-driven *SlANT1* expression can enhance gray mold resistance and shelf life of tomato fruit.

### *pSlPR10*-driven transgenic expression of *SlMYB31* produces tomato fruit with delayed water loss

To further showcase the utility of *pSlPR10* in genetic engineering of tomato fruit quality, we harnessed it for expressing *SlMYB31*, which has been shown to positively regulate the biosynthesis of very-long-chain fatty acids and cuticular wax in tomato [41]. We speculated that targeted *SlMYB31* expression in the exocarp could help to reduce the water loss of tomato fruit by thickening the cuticular wax in the skin. To this end, transgenic tomato cv. AC plants expressing *pSlPR10::SlMYB31* were generated. As determined by RT-qPCR, *pSlPR10*-driven *SlMYB31* expression in the exocarp of transgenic plants led to a 15- to 18-fold increase in its transcript levels relative to WT plants (Fig. 5a). When grown in a greenhouse under natural light conditions, these transgenic plants, including their fruits, exhibited a wild-type appearance (Fig. 5b; Fig. S10, see online supplementary material). Since toluidine blue staining provides an approach to evaluate the water permeability of tomato fruit, we stained transgenic and WT fruits at 30 dpa with toluidine blue, which led to a much smaller area of staining in the former than that in the latter (Fig. 5b). These results indicated a negative correlation between the expression levels of *SlMYB31* in the exocarp and the water permeability of fruits. To substantiate that the reduced water permeability of transgenic fruit was caused by SlMYB31-stimulated waxy thickening in the exocarp, frozen sections of the exocarp from tomato fruits at 30 dpa were prepared using a cryostat and stained by the Sudan III dye. Indeed, microscopic observation revealed a significantly thicker waxy layer in the exocarp of transgenic fruits than WT fruits (Fig. 5c and d).

We next investigated whether targeted *SlMYB31* expression in the exocarp could affect the fruit shelf life. Storage tests for fruits harvested at 7 dpb uncovered that transgenic fruits showed slower water loss, delayed fruit softening and collapse during the 7-week storage period when compared to WT fruits (Fig. 5e–g). These results suggested that *pSlPR10*-driven *SlMYB31* expression is able to extend the fruit shelf life by thickening the cuticular wax to delay water loss. As far as we know, this is the first report of fruit wax thickening using an exocarp- or peel-specific promoter.

### Three tomato exocarp-preferential promoters exhibit coincided activities in the gynophore of *Arabidopsis*

It was reported that several promoters from *Arabidopsis* (*Arabidopsis thaliana*) can maintain their expression specificities in counterpart tissues of tomato [23]. Inversely, the tomato *RIP1* promoter with maximum activity in ripe fruit can drive highest *GUS* expression in *Arabidopsis* silique [20], which develops from the fertilized gynoecium and can be functionally regarded as a fruit for seed dispersal [42]. Because *Arabidopsis* obviously lacks an exocarp, we were curious about the tissue specificity of *pSlPR10*-controlled transgene expression in *Arabidopsis*. Therefore, transgenic *Arabidopsis* plants expressing *pSlPR10::GUS* were generated. Interestingly, we found that the GUS activity occurred strongly in the gynophore (Fig. 6a), the basal-most region of silique, though the GUS activity could also be detected in the anthers but not in the leaves of *Arabidopsis* (Fig. 6a). These findings hinted at a compelling possibility that tomato exocarp and *Arabidopsis* gynophore are evolutionarily equivalent, especially considering the immediate physical adjacency of gynophore to silique (fruit) in *Arabidopsis*.

To obtain additional evidence for this hypothesis, we generated transgenic *Arabidopsis* plants expressing *GUS* under the control of a 2272-bp *SlABCG10* promoter or 2213-bp *SlHH3* promoter, as *SlABCG10* and *SlHH3* were also among the top three exocarp preferentially expressed genes, despite at lower expression levels than *SlPR10* (Fig. 1d). Indeed, GUS staining was mainly observed in the gynophore of transgenic *Arabidopsis* plants in both cases (Fig. 6b and c). A weak activity of the *SlABCG10* promoter was also detected in the stigma of *Arabidopsis* (Fig. 6b). Taken together, these results may provide a clue of evolutionary homology between tomato exocarp and *Arabidopsis* gynophore, though future studies are still needed to fully testify this hypothesis. To our knowledge, this is an unprecedented report on the *Arabidopsis* gynophore-preferential promoters, which will be useful for gene functional study in this tissue in the future.

## Discussion

Isolation and characterization of novel tissue-specific promoters is of prominent significance for genetic engineering and synthetic biology in plants. In contrast to constitutive promoters, tissue-specific promoters allow targeted gene expression in intended tissues or cell types, thereby minimizing unnecessary resource consumption, genetic perturbation, and potential growth inhibitory effects at a whole plant level. In tomato, an exocarp-specific promoter is in demand. This is not only because the exocarp plays a critical role in determining the fruit appearance, stress resistance, and shelf life [26, 27], but also because this tissue can supply potentially useful chassis cells for synthetic biology to produce high-value compounds, which later can be extracted from recycled non-edible tomato peel wastes.

In this study, we identified tomato *SlPR10* as an exocarp preferentially expressed gene and applied its promoter (*pSlPR10*) for exocarp-based genetic engineering of anthocyanin or cuticular wax biosynthesis, which conferred gray mold resistance or reduced water loss and prolonged shelf life to tomato fruit. Our work addressed the research gap concerning tomato exocarp-specific promoters and provided proof of concept for how such a promoter can be useful in improving tomato fruit quality. Serendipitously, *pSlPR10* was found to direct targeted transgene expression in *Arabidopsis* gynophore (Fig. 6a). In the oil-bearing crop peanut (*Arachis hypogaea L.*), the gynophore plays a vital role in peanut seed production by downward elongating to sow the fertilized ovule into the soil. Intriguingly, *AhPR10*, a homologous gene of *SlPR10*, is also highly expressed in peanut gynophore [43]. It will be inviting in the future to explore whether *pSlPR10* can be applied for genetic engineering of gynophore in peanut and *Brassica* crops.

Anthocyanins, as versatile plant stress mitigators and human health-promoting antioxidants [44], are found in the skin of many fruits such as cherry, blueberry, and eggplant. However, domesticated tomato cultivars seldom contain anthocyanins in fruit, let alone the skin. An exception is the tomato variety Indigo Rose (InR), which was generated through introgression breeding [40] to express *SlANT2-like*, a homologous gene of *SlANT1*, in a light-inducible manner [39]. Accordingly, anthocyanins are only accumulated in the part of exocarp exposed to sunlight [45]. Meanwhile, the amounts of anthocyanin accumulation in the exocarp of InR fruits appear to be negatively correlated with the fruit sizes [40]. By genetic engineering, Bassolino and colleagues applied the light-inducible fruit-specific promoter *PLI* to express *Rosea 1*, another homologous gene of *SlANT1*, to render anthocyanin enrichment in the exocarp [27]. However, similar to the InR fruit, transgenic *PLI::Rosea1* fruit also showed uneven purple coloration of the skin depending on the sunlight. Moreover, the *PLI* promoter-mediated expression starts in the exocarp at immature stages but gradually spreads inward to the entire fruit during ripening [22]. Unlike the *SlANT2-like* or *PLI* promoter [22, 39], the *SlPR10* promoter was not induced by light (Fig. S7c and d, see online supplementary material). Accordingly, our transgenic *pSlPR10::SlANT1* fruits displayed even purple coloration on the skin independent of light exposure (Fig. 3c and d; Figs S6a and b, S7a and b, see online supplementary material). We also did not detect a dramatic difference in fruit sizes between transgenic *pSlPR10::SlANT1* fruits and WT fruits (Fig. 3i; Fig. S8b, see online supplementary material).

Moderate levels of anthocyanins could be seen in the leaves of transgenic *pSlPR10::SlANT1* plants (Fig. 3g;Figs. S5 and S8a, see online supplementary material). This was likely due to the background activity of *pSlPR10* in leaves, which allowed weak but persistent production and accumulation of anthocyanins over time. Of note, none of the transgenic tomato plants showed obvious growth retardation under our experimental conditions (Fig. 3g and h;Figs S5 and S8a, see online supplementary material). It should be emphasized that stringent fruit specificity is rather difficult to achieve. Most of the purported tomato fruit-specific promoters, despite being predominantly active in fruit, also showed detectable expression in non-fruit tissues [15, 16, 19–24]. In particular, even for the frequently used ripening-specific *E8* promoter, transgene expression in unexpected tissues has occasionally been noticed [22, 46]. Based on the predominant activity of *pSlPR10* in the exocarp over the stem and leaves, this promoter should be sufficiently effective for most applications requiring a considerable degree of exocarp specificity. Nevertheless, two strategies may be considered for further optimizing the exocarp specificity of *pSlPR10*. One strategy is to map the core region of *pSlPR10* responsible for the exocarp specificity by promoter deletion analysis and then fuse it with the *35S* minimal promoter to obtain an enhanced exocarp-specific promoter. Such a strategy has been employed to improve several fruit-specific promoters, such as the *2A11* and *SlHDC* promoters [16, 47]. The other strategy is to leverage the CRISPR/Cas9 technology-mediated promoter editing to introduce saturated indel mutations throughout *pSlPR10* to screen for a promoter variant with enhanced exocarp specificity, as demonstrated by several successful studies in crops [48–50].

We envision that future metabolic engineering or molecular farming in the exocarp of tomato fruit can be facilitated by using *pSlPR10*. When several endogenous genes need to be simultaneously activated in the exocarp, it is tempting to use *pSlPR10* to drive the expression of dCas9-TV, a guide RNA (gRNA)-directed artificial transcriptional activator [51], which in turn can activate the transcription of multiple target genes from their endogenous genomic loci via promoter-bound gRNAs [52]. Conceivably, concurrent transcriptional activation of *SlANT1* and *SlMYB31* by dCas9-TV in the exocarp may be able to further extend the fruit shelf life via combinatorial effects of anthocyanin accumulation and waxy thickening. In addition to endogenous genes, *pSlPR10* can be applied to express exogenous genes encoding metabolic enzymes in the exocarp to produce non-native high-value compounds, such as betalains [53] and astaxanthin [54], which promises new opportunities for quality improvement of tomato fruit.

## Conclusion

In this study, we identified by RNA-seq and RT-qPCR analyses that the tomato *SlPR10* gene was abundantly and predominantly expressed in the exocarp. We further demonstrated that a 2,087-bp *SlPR10* promoter can be very useful for exocarp-based genetic engineering of tomato fruit quality. By taking advantage of this promoter,transgenic expression of *SlANT1* encoding a master regulator of anthocyanin biosynthesis produced purple-skinned tomato fruits, which showed enhanced resistance to the gray mold disease, delayed fruit softening and extended shelf life.Exocarp-based expression of *SlMYB31* encoding a master regulator of wax biosynthesis delayed fruit softening and water loss and extended fruit shelf life. 

## Materials and methods

### Plant materials and growth conditions

The tomato (*S. lycopersicum*) AC or Micro-Tom cultivar and *Arabidopsis* (*A. thaliana*) ecotype Col-0 were used as wild-type plants in this study. Tomato seeds were soaked in water until germination. Tomato seedlings were then transferred to the Jiffy soil (Jiffy Group, Zwijndrecht, Netherlands) and grown under photoperiods of 16 h light (75 μmol/m^2^/s) at 23°C and 8 h dark at 21°C. Alternatively, tomato seedlings were grown in a greenhouse under natural sunlight at temperatures ranging from 20–26°C. After stratification at 4°C for 2 days, *Arabidopsis* seeds were germinated on the Jiffy soil in a plant growth room under photoperiods of 16 h light (75 μmol/m^2^/s) at 23°C and 8 h dark at 21°C.

### Molecular cloning and plant transformation

The sequences of *pSlPR10* and terminator and the coding sequence of *SlANT1* were obtained from the AC tomato genome by PCR. The *GFP* and *GUS* reporter genes were retrieved from existing plasmids in the laboratory. The *SlMYB31* gene was cloned by RT-qPCR. The *pSlPR10*-based expression cassettes were constructed into the pCAMBIA binary vector using the ClonExpress MultiS One Step Cloning Kit (Vazyme Biotech Co., Ltd., Nanjing, China). The resulting binary plasmids were transformed into *Agrobacterium tumefaciens* strain GV3101 cells by electroporation. Agrobacteria containing the verified plasmid were used for transforming tomato cotyledons. Transformed tomato cotyledons were selected by hygromycin resistance and regenerated into whole plants according to a standard protocol [55]. Transgenic plants were validated by Sanger sequencing of the genomic PCR products spanning transgenes. The primers used in PCR are listed Table S1 (see online supplementary material).

### RNA-seq and RT-qPCR

The exocarp or mesocarp of mature green (30 dpa) or red ripe (7 dpb) tomato fruits or pooled leaves, stems, and roots from both developmental stages were ground in liquid nitrogen into powder. Total RNA was extracted from tissue powder using 1 ml RNAiso Plus reagent (Takara, Beijing, China) according to the manufacturer’s instructions. RNA-seq for samples with two biological replicates and data analyses were carried out by the BioMarker company (Beijing, China) as previously described [56]. For RT-qPCR, total RNA of 1 μg per sample was converted into first-strand cDNA using the PrimeScript RT Reagent Kit with gDNA Eraser (Takara, Beijing, China). The qPCR was performed in a LightCycler 96 Instrument (Roche, Shanghai, China) using TB Green Premix Ex Taq (Takara, Beijing, China). *SlACTIN* was used as a reference gene. The primers used in qPCR are listed in Table S1 (see online supplementary material).

### Detection of GFP fluorescence

To detect the GFP fluorescence emitted from a whole plant, a LUYOR-3415RG dual-wavelength fluorescence protein excitation flashlight (LUYOR, Shanghai, China) was used to illuminate the plant and images were captured by a DSLR camera with filter lens. To detect the GFP fluorescence emitted from a fruit section, tomato fruit was sliced into 3-mm-thick sections using a blade and GFP fluorescence was observed using the Gelview 6000plus imaging system (Biolight Biotechnology, Guangzhou, China). For the GFP fluorescence emitted from the exocarp cells, a DMi8 fluorescence inverted microscope (Leica, Shanghai, China) was used for observation and imaging.

### Fruit storage test

Fruits were harvested at 7 dpb and surface sterilized by 75% ethanol for 1 min, followed by rinsing in sterile water and air-drying. Each fruit was placed in a sterilized jar and kept at room temperature in the dark. The fresh weight of each fruit was measured and the visual softening and collapse of the fruit were assessed every week.

### *B. cinerea* infection

*B. cinerea* strain 2100 was grown and the spores were collected as described previously [57]. Fruits were harvested at 7 dpb and surface sterilized. The fungal culture was diluted with the Vogel buffer to 1 × 10^6^ spores ml^−1^ and incubated at 28°C for 1.5 h before inoculation. The surface of each fruit was incised to create a 1-cm long and 1-mm deep cross-shaped wound. The spore liquid of 5 μl was added to the center of the wound and the challenged fruits were grown in the dark. Lesion diameter was measured 72 h after inoculation. To quantify *B. cinerea* growth, infected fruit exocarp tissues of 1 cm^2^ around the wound site were harvested and qPCR analysis was performed using the primer pairs of *BC-Cutin* and *Sl-Actin* (Table S1, see online supplementary material) to determine the fungal biomass and tomato biomass, respectively. Total DNA isolation and qPCR were conducted as previously described [36].

### Anthocyanin measurement and TEAC assay

Anthocyanins were extracted from tomato exocarp or mesocarp at the mature green or red ripe stage as described previously [38]. Briefly, fruit peels of 0.1 g was ground to powder and resuspended in 1 ml extraction buffer containing 18% 1-propanol, 1% HCl, and 81% water. Absorbance values (A535 and A650) of the supernatant were determined using a microplate reader (Varioskan LUX, Thermo Fisher, Shanghai, China). The anthocyanin content was measured as (A535-A650)/g fresh weight and further expressed as mg/g fresh weight based on the standard curve generated using petunidin-3-(p-coumaryl)-rutinoside-5-glucoside (Caobenyuan Co., Ltd, Nanjing, China) as a reference standard. For the TEAC assay, anthocyanins were extracted from fruits at 7 dpb using 5 ml of acidified (0.3% HCl, vol/vol) methanol for 24 h at 4°C as described earlier [44]. After extraction, samples were centrifuged for 20 min at 5000 *g*. Anthocyanins in the supernatant were determined by using the Total Antioxidant Capacity kit (Grace Biotechnology, Suzhou, China) according to the manufacturer’s instructions. Results were presented as TEAC in mmol of Trolox per kg of fresh weight.

### Toluidine blue staining

Toluidine blue (Sigma-Aldrich, Shanghai, China) was dissolved in water to a concentration of 5%. Fruits were placed in a shallow container with approximately half of the fruit surface submerged in the toluidine blue solution. After 4 h staining, the toluidine blue solution was removed and fruits were washed gently with water to remove excess dye before photographing.

### Measurements of cuticle wax thickness

Fruit slices were generated based on the protocol described earlier [58]. Cubes of 3 mm side from the equatorial pericarp of each fruit were cut using a razor blade and immediately immersed in the FAA fixative containing 5% formalin, 5% glacial acetic acid, 50% ethanol, and 45% distilled water. After fixation overnight at 4°C, the sample with a thickness of 10 μm was obtained using a cryostat (Leica CM1950, Shanghai, China). Sudan III powder (Sangon, Shanghai, China) of 0.5 g was dissolved in 50 ml ethanol, mixed well, and filtered through a syringe filter to remove precipitates. The sample slice of 10 μm was incubated with the staining solution for 10 min, and then rinsed sequentially with 50% alcohol and distilled water before being observed under a DMi8 fluorescence inverted microscope (Leica, Shanghai, China). Thickness of the stained waxy layer was determined as the average of 15 measurements from five biological replicates.

### Evaluation of the induction of *pSlPR10* by light or *B. cinerea* infection

To evaluate the effect of light exposure on the activity of *pSlPR10*, protoplasts were isolated from cotyledons of seven-day-old transgenic *pSlPR10::GFP-GUS* tomato seedlings according to the reported protocol [59]. Protoplasts were then divided into two equal aliquots, one incubated under the light for 6 hr and the other in the dark. Total proteins were extracted from pelleted cells by boiling in SDS loading buffer and resolved by SDS-PAGE analysis. GFP-GUS abundances were determined sequentially using the Rabbit polyclonal anti-GFP antibody (A01704, GenScript, Nanjing, China) and the anti-rabbit IgG antibody (7074S, Cell Signaling Technology, Shanghai, China) at 1:10000 dilution. To evaluate the effect of *B. cinerea* infection on the activity of *pSlPR10*, the fungal spore suspension of 5 μl (2500 spores) was added onto the detached leaves of transgenic *pSlPR10::SlANT1* plants for 6 h. Three leaf discs with a diameter of 4-mm around the inoculation site were collected for SDS-PAGE and western blot analyses using the anti-FLAG antibody (A2220, Sigma-Aldrich, Shanghai, China) at 1:10000 dilution.

### GUS staining

GUS staining was conducted using a specialized GUS staining kit (Huayueyang Biotechnology, Beijing, China) according to the instructions provided by the manufacturer. Briefly, an appropriate amount of prepared GUS staining working solution was added to cover the tissues for staining. After incubating at 37°C for 4 h in the dark, the chlorophyll of the samples was removed by immersing the samples in 70% ethanol until decolorized. Sample photographing was conducted using a S8APO stereomicroscope (Leica, Shanghai, China).

### Statistical analysis

Statistical analysis of the experimental data in this study was performed using two-tailed Student’s *t*-test or two-way ANOVA with Tukey’s multiple comparisons test. For all tests, *P* values of <0.05 were considered statistically significant.

## Acknowledgements

This work was supported by grants from the National Key Research and Development Program of China (2019YFA0906202) and National Natural Science Foundation of China (32125004) to J.-F.L. The authors thank members of the Li laboratory for stimulating discussion on this work.

## Author contributions

J.-F.L. conceived and supervised the study. X.-M.R. performed all experiments. X.-M.R., X.X., and J.-F.L. analysed the data. J.-F.L. and X.-M.R. wrote the manuscript. All authors approved the final version of the manuscript.

## Data availability

The RNA-seq data reported in this paper can be found at the Gene Expression Omnibus (GEO) with the accession ID GSE235023.

## Conflict of interest statement

Based on the data of the current research, a China invention patent (ZL202210684553.8) has been granted to the authors.

## Supplementary data

Supplementary data is available at *Horticulture Research* online.

## Supplementary Material

Web_Material_uhae035
