# Supplementary material for: Identification and application of an exocarp-preferential promoter for genetic engineering of tomato fruit
Source: Hortic Res. 2024 Jan 31;11(3):uhae035. doi: 10.1093/hr/uhae035 (PMC10967692; doi:10.1093/hr/uhae035)
Supplement: Web_Material_uhae035 [file web_material_uhae035.zip › Supplementary Data.pdf]

Supplementary Data

Identification and application of an exocarp-preferential promoter for genetic engineering of tomato fruit

Xue-Ming Ruan[1], Xiangyu Xiong[1], Jian-Feng Li[1,*]

[1]Guangdong Provincial Key Laboratory of Plant Resources, State Key Laboratory of Biocontrol, School of Life Sciences, Sun Yat-Sen University, Guangzhou 510275, China

*Correspondence: lijfeng3@mail.sysu.edu.cn

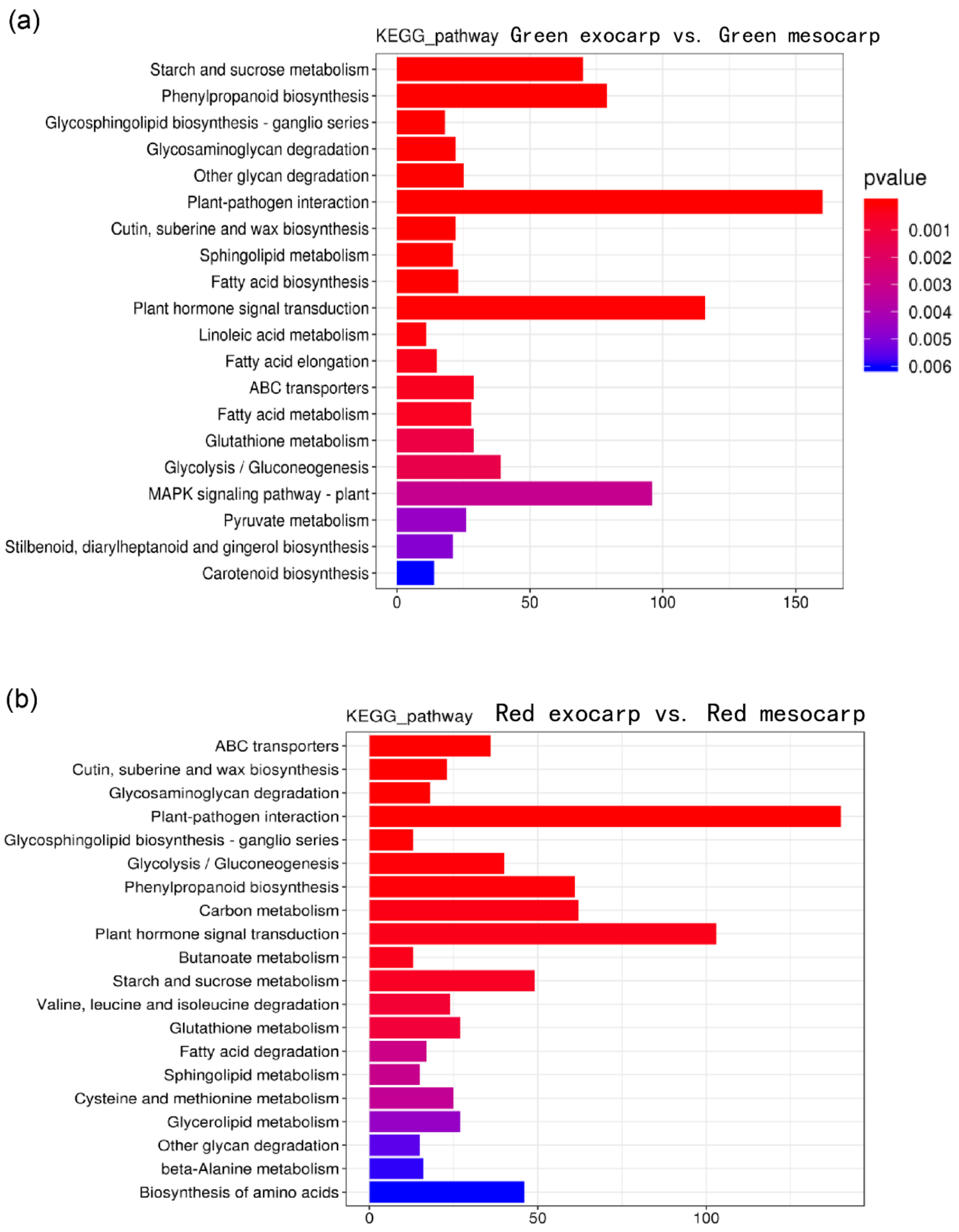

Figure S1. KEGG analysis indicates that differentially expressed genes between the exocarp and mesocarp at the mature green (**a**) or red ripe (**b**) stage are overrepresented by those with potential roles in plant-pathogen interactions. The X-axis means the gene numbers, while the Y-axis lists the pathway categories. The color of the pathway bar indicates *P* value of the hypergeometric test by referring to the color scale. Green, 30 days post-anthesis. Red, 7 days post-breaker.

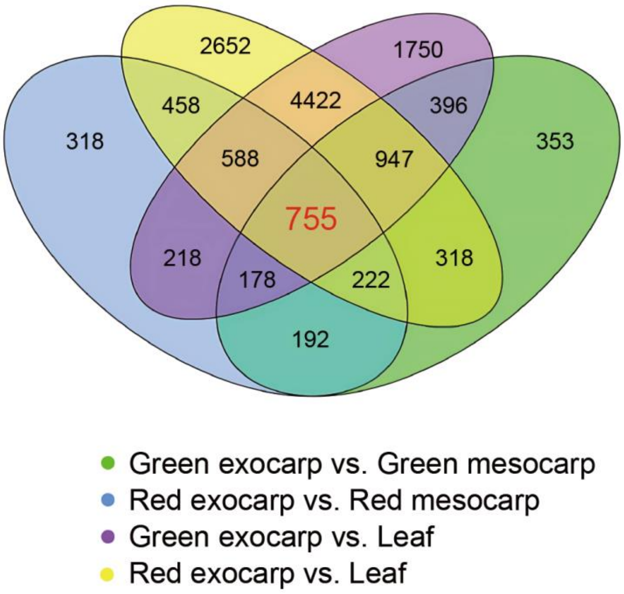

Figure S2. An overlapping subset of 755 genes are differentially expressed in the exocarp compared to the mesocarp and pooled leaves in tomato. Green, 30 days post-anthesis. Red, 7 days post-breaker. Pooled leafs were collected from both developmental stages.

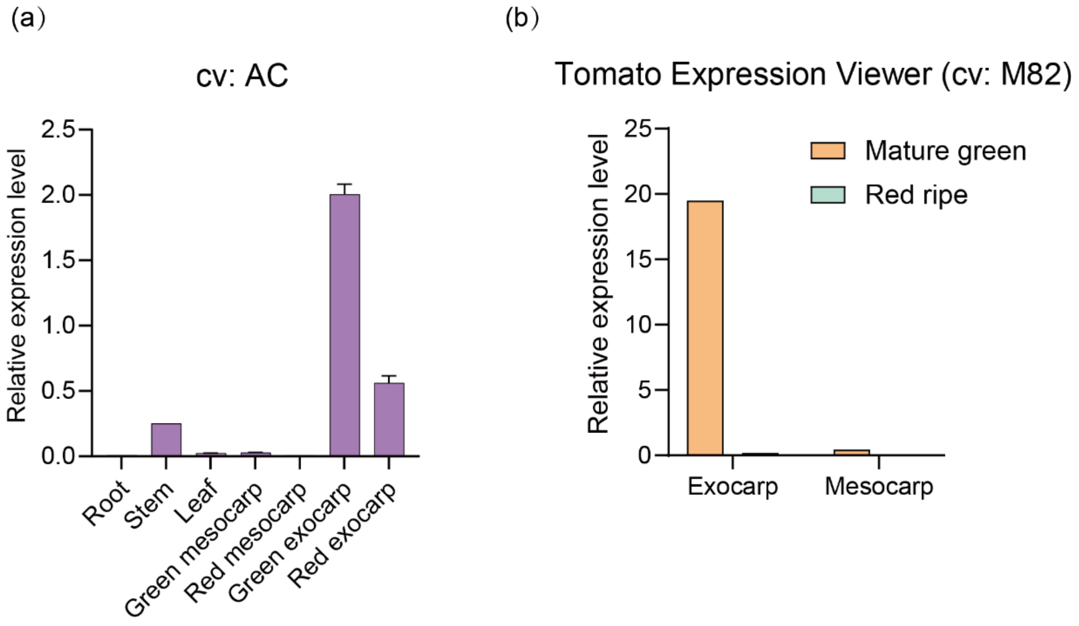

Figure S3. Exocarp-preferential expression pattern of *SlPR10* in different tomato cultivars. **a**, Exocarp-preferential expression pattern of *SlPR10* in tomato cv. AC revealed by RT-qPCR analysis. Data are shown as means ± SD of three biological replicates. *SlACTIN* was used as a reference gene to normalize the relative expression level. **b**, Exocarp-preferential expression pattern of *SlPR10* in the tomato cv. M82 fruit shown by the Tomato Expression Viewer. *SlACTIN* (Solyc03g078400) was used as a reference gene to normalize the relative expression level. This database can be accessed at https://tea.solgenomics.net/expression_viewer/input.

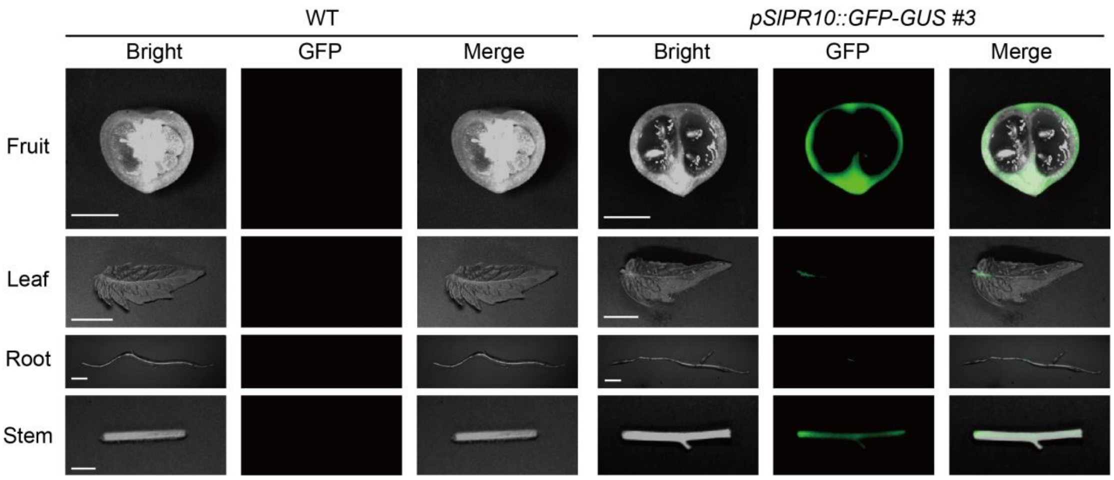

Figure S4. *pSlPR10* enables exocarp-preferential expression of the *GFP-GUS* reporter in transgenic tomato cv. Micro-Tom. Transgenic plants were examined using a fluorescence imaging system and representative images for mature green fruit (cross-section), leaf, root, and stem are shown. Scale bar = 1 cm.

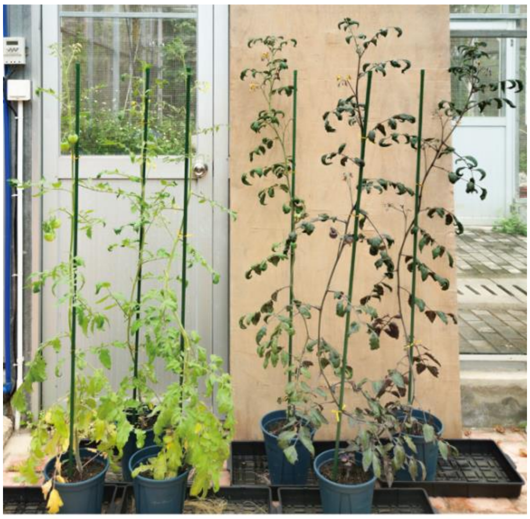

Figure S5. Transgenic AC plants expressing *pSlPR10::SlANT1* display comparable growth as WT plants in a greenhouse under natural light conditions. Two-month-old plants are shown.

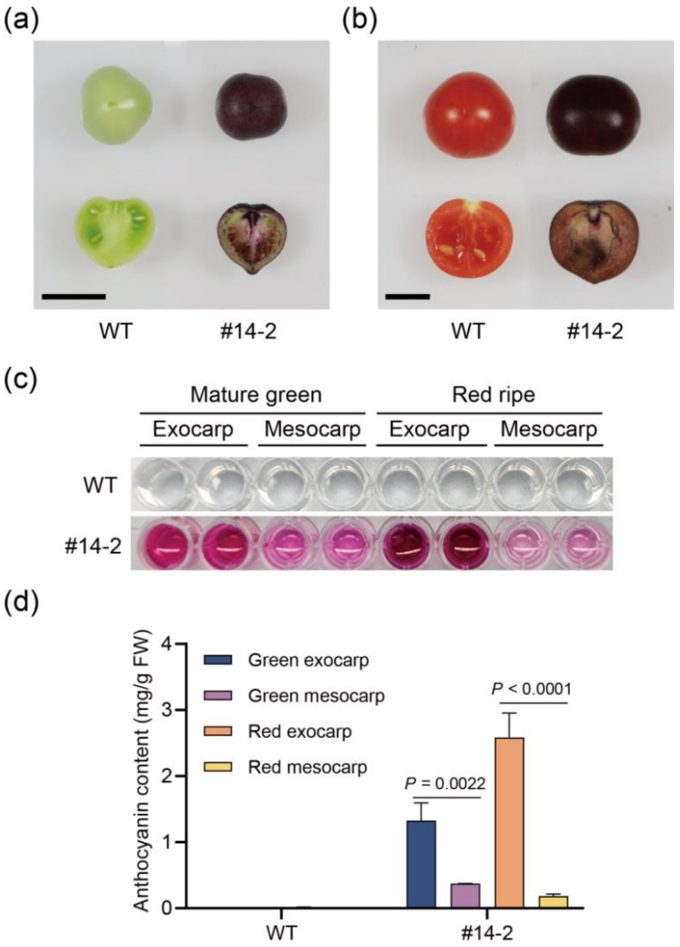

Figure S6. Transgenic Micro-Tom fruits expressing *pSlPR10::SlANT1* display anthocyanin overaccumulation in the exocarp. **a**,**b** The skin of transgenic fruits at the mature green (**a**) or red ripe (**b**) stage exhibited a dark purple appearance. Scale bar = 1 cm. **c**,**d** Anthocyanin overaccumulation in the exocarp of transgenic fruits seen by naked eyes (**c**) or measured based on absorbance values (**d**). The anthocyanin content was measured as (A535-A650)/g fresh weight and further expressed as mg/g fresh weight according to the standard curve generated using petunidin-3-(p-coumaryl)-rutinoside-5-glucoside as a reference standard. Data are presented as means ± SD (n = 3). Statistical analysis was conducted using two-tailed Student's *t*-test.

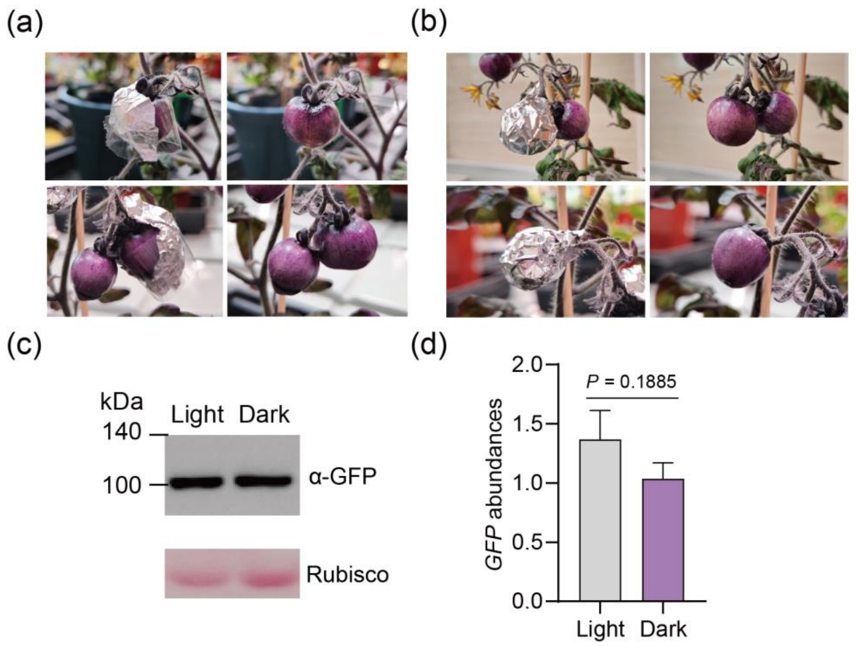

Figure S7. The activity of *pSlPR10* is not significantly induced by light. **a**,**b** Shading (**a**) or complete darkness (**b**) treatment for 10 days failed to affect the degree of purple coloration on transgenic Micro-Tom fruits expressing *pSlPR10::SlANT1*. The images of fruits wrapped with aluminum foil indicate how the treatment was conducted. **c**,**d** Light illumination failed to dramatically induce *pSlPR10::GFP-GUS* expression. In (**c**), equal aliquots of mesophyll protoplasts isolated from transgenic AC plants expressing *pSlPR10::GFP-GUS* were subjected to light or dark treatment for 6 h and *GFP-GUS* expression was determined by immunoblot using the anti-GFP antibody. Rubisco serves as a loading control. In (**d**), immunoblot and Rubisco signals were quantified by Image J and their ratios were used to indicate GFP-GUS abundances. Data are presented as means ± SD of three biological replicates. Statistical analysis was conducted using two-tailed Student's *t*-test and no significance was observed.

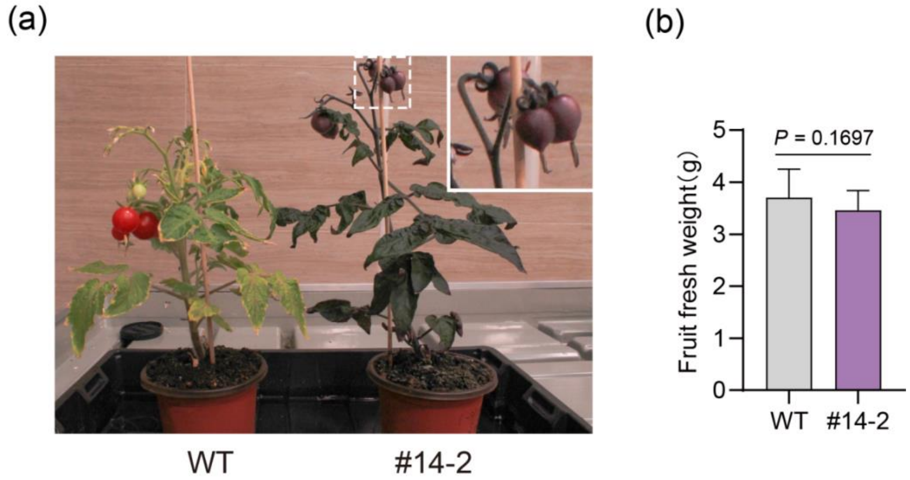

Figure S8. Transgenic Micro-Tom plants expressing *pSlPR10::SlANT1* display comparable growth (**a**) and fruit weights (**b**) as WT plants in a greenhouse under natural light conditions. The inlet image in (**a**) shows a magnified view of the boxed region in the transgenic plant. In (**b**), data are presented as means ± SD (n = 15). Statistical analysis was conducted using two-tailed Student's *t*-test and no significance was observed.

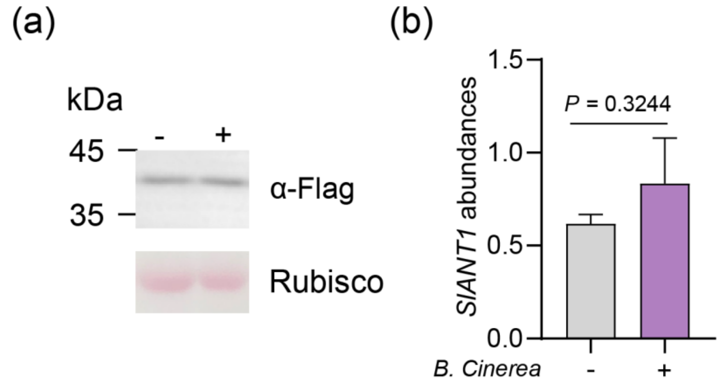

Figure S9. The activity of *pSlPR10* is not significantly induced by *B. cinerea* infection. **a** *B. cinerea* infection failed to dramatically induce *SlANT1* expression in leaves of transgenic AC plants expressing *pSlPR10::SlANT1*. Detached leaves were treated with 2,500 spores of *B. cinerea* (+) or mock (-) for 6 h. *SlANT1* expression was determined by immunoblot using the anti-FLAG antibody. Rubisco serves as a loading control. **b** Quantification of the data shown in (**a**). Immunoblot and Rubisco signals were quantified by Image J and their ratios were used to indicate SlANT1 abundances. Data are presented as means ± SD of three biological replicates. Statistical analysis was conducted using two-tailed Student's *t*-test and no significance was observed.

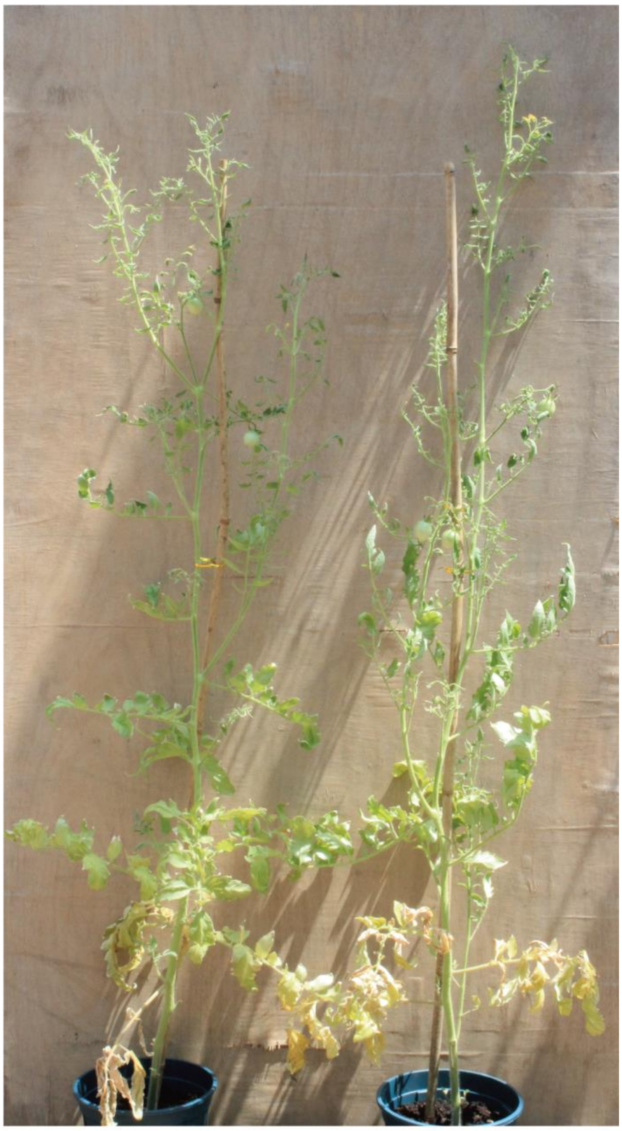

WT pSlPR10::SlMYB31 #5-2

Figure S10. Transgenic AC plants expressing *pSlPR10::SlMYB31* display comparable growth as WT plants in a greenhouse under natural light conditions. Two-month-old plants are shown.

Table S1 Primers used in this study

Primer name	Primer （5' to 3'）
pSlPR10	F: GGCTTTAGTCAAGATTTCATCAACTCCATGA<br>R:CATAATGTTTGATTAAGTATTTATATTTGAAAGAAAAAAAGATATGA
pSlABCG10	F: TTCAATTTTAAAGGAAGTGGATCACTTTGAGAG<br>R: TTATCGCGGTTGGTTAGAC
pSlHH3	F: TATTAGGCTGTAGTGTGGAGGTTTACATAGG<br>R:GTTTTTGTTTCTTCTGAATCTGTTAAATATACAAATTATATAATGG
GUS	F: ATGTTACGTCCTGTAGAAACCC<br>R:CCAAATGTTTGAACGATCTTATTGTTTGCCTCCCT
GFP	F: ATGGTGAGCAAGGGCGAGGA<br>R: CTTGTACAGCTCGTCCATGCC
SlMYB31-2HA	F: ATGGGAAGGCCACCTTGC<br>R:CCAAATGTTTGAACGATCTCATGCATAGTCAGGAACATCATATGGGTATGCATAGTCAGGAACATCATATGGGTATCCAAAAAAGTCAGCAGATTCACCCAAAC
SlANT1-2FLAG	F: ATGAACAGTACATCTATGTCTTCATTGGG<br>R:CCAAATGTTTGAACGATCTTACTTGTCATCATCATCTTTATAATCCTTGTCATCATCATCTTTATAATCTCCATCAAGTAGATTCCATAAGTCAATTTCAGCAG
ABCG10-qPCR （*Solyc04g070970*）	F: CCTGGACCTAGTGTTGCCTG<br>R: CCAAACCTAAGCCCTGAGCA
BCAD-qPCR （*Solyc06g059840*）	F: CACATGTCAAGAACCAGAGGC<br>R: AAACAATTCTGTCAATGCAGGCT
FP-qPCR （*Solyc01g105620*）	F: TACCAATTGTCCCACGTGCC<br>R: GTCTCCGCCCTGAAATCTGT
HH3-qPCR （*Solyc05g032680*）	F: GGACAGAAAGTTTAAGTGGGAACC<br>R: GTAGCCCCAACGATCGTCAT
IQM2-qPCR （*Solyc01g005800*）	F: GCAGTTTCTCGTTGGTCGAG<br>R: TGTCGTGGGTCGATAGCTTCA
KIN5D-qPCR （*Solyc09g007030*）	F: AAGTTACCTCTGAAGTTCTCCTCAACT<br>R: TTGCAGCCAGCTCCTCAAACT
LECRK4-qPCR （*Solyc09g075910*）	F: TGTCTGGGGACTTTGCCTTC<br>R: TCAGCTGGATTGTCGCGATT
SNL6-qPCR （*Solyc06g061280*）	F: ACGCTTCCACTCGATTAGGC<br>R: ACGACGACTCGCCATGATTT
STK-qPCR （*Solyc08g061250*）	F: GCCGGAAGGGGATGATACTC<br>R: TCATTGTCGTCAAATGACCTGT
TSI1-qPCR （*Solyc09g090990*）	F: TCAAGTACTTTATTGGACCACCTTC<br>R: TCAAGTACTTTATTGGACCACCTTC
WSD1-qPCR （*Solyc01g011430*）	F: AGCTGTCGGAATTCAGGCTT<br>R: AATCCAATTCCCCCATTTTGCC
PR10-qPCR （*Solyc09g090980*）	F: ATGAACTTTGTTGAAGGTGGAC<br>R: GGCAATTGATTCCAATTTGTCACC
NB-ACT-qPCR	F: CTTGAAACAGCAAAGACCAGC<br>R: CATCCTATCAGCAATGCCCG
SlACTIN-qPCR	F: TTGCTGACCGTATGAGCAAG<br>R: GGACAATGGATGGACCAGAC
Sl-Actin	F: ACAACTTTCCAACAAGGGAAGAT

（*LOC101262163*）	R: TGTATGTTGCTATTCAG GCTGTG
BC-Cutin	F: ATTCCACAATATGGCATGAAATC<br>R: ATGTTATCTC ATGTTATCTC
